# Cavities and Calories: Exploring the Relationship Between Early Childhood Caries and Body Mass Index

**DOI:** 10.7759/cureus.93755

**Published:** 2025-10-03

**Authors:** Surbhi Sharma, Meenakshi Sharma, Tripti Sharma, Abhishek Haldiya, Kamal Soni, Hage Monju

**Affiliations:** 1 Department of Pedodontics and Preventive Dentistry, Rajasthan University of Health Sciences College of Dental Sciences, Jaipur, IND; 2 Department of Pediatric Dentistry, Rajasthan University of Health Sciences College of Dental Sciences, Jaipur, IND; 3 Department of Pediatric Dentistry, Government College of Dentistry, Indore, IND

**Keywords:** body mass index (bmi), child nutrition, early childhood caries (ecc), pediatric preventive dentistry, underweight children

## Abstract

Background: Early childhood dental health is a key determinant of overall health and well-being. Healthy dentition ensures proper mastication, clear speech, and acceptable appearance, all of which contribute to a child's nutrition, communication, and social development. In recent years, growing attention has been directed toward the association between dental caries and children's physical growth. Body mass index (BMI), widely recognized as an indicator of nutritional status and growth patterns, has been explored in this context. Aberrant BMI values, whether indicative of undernutrition or overweight, may influence oral health outcomes, while conversely, dental caries can impair dietary intake and thereby affect BMI and growth trajectories in early childhood.

Aim: This study aimed to explore the association between early childhood caries and growth status, measured by BMI, in children aged 3-6 years.

Methodology: A cross-sectional study was conducted among 260 children attending a pediatric dental outpatient department. A structured questionnaire was completed by parents, and clinical examinations were performed according to the World Health Organization (WHO) guidelines. Anthropometric measurements were taken, and BMI-for-age percentiles were calculated using the Centers for Disease Control and Prevention (CDC) growth charts. Children were categorized as underweight, normal weight, overweight, or obese.

Result: The prevalence of early childhood caries in the study population was 34.2%. A significantly higher proportion of early childhood caries was observed among underweight children (49.4%) compared to those with normal or higher BMI (p=0.001). No significant association was found between early childhood caries and age or gender.

Conclusion: A significant association was identified between early childhood caries and underweight status in preschool-aged children. These findings underscore the need for integrated oral health and nutrition strategies to support optimal growth and development in early childhood.

## Introduction

Early childhood dental health is crucial for the overall well-being and development of young children [1]. Establishing proper oral hygiene habits during infancy sets the foundation for sustained oral health throughout childhood and adolescence and into adulthood [2]. Dental caries is one of the most common chronic diseases among children globally and presents a significant burden on healthcare systems due to its treatment demands and potential complications [1].

The status of primary teeth has a strong correlation with the health of permanent dentition, underscoring the importance of identifying and addressing risk factors for caries in early childhood [3]. The American Academy of Pediatric Dentistry (AAPD) defines early childhood caries (ECC) as the presence of one or more decayed (non-cavitated or cavitated), missing (due to caries), or filled surfaces in any primary tooth in a child under 71 months of age. Severe ECC is characterized by more extensive involvement, such as smooth surface lesions or decay in anterior teeth, depending on the child's age [4].

ECC is a significant public health issue affecting infants and preschool-aged children worldwide [5]. In India, various studies report ECC prevalence ranging from 25% to 70%, with particularly high rates observed in southern regions [6]. The burden is even greater in disadvantaged populations of both developing and industrialized countries, where undernutrition is also a prevalent concern [7].

ECC is understood to have a multifactorial etiology, influenced by biological, environmental, and socio-behavioral determinants. Psychosocial factors, including maternal mental health, low educational attainment, and poverty, further increase the likelihood of caries in early childhood [8]. Dietary behaviors, widespread consumption of sugary snacks and drinks between meals, have been strongly associated with ECC development [5].

In recent years, there has been growing interest in the association between dental health and physical growth in children. Body mass index (BMI) is a widely used metric for assessing nutritional and growth status, calculated from height and weight. It is typically expressed in kg/m² and is applicable in children over 24 months of age [9,10]. BMI is both age- and gender-specific and can be converted into standardized z-scores for cross-age comparisons [10].

Despite extensive global research, there is a lack of localized data on the prevalence of ECC and its association with other factors in the Jaipur district of Rajasthan. Therefore, this study was designed to evaluate the relationship between ECC, BMI, and socioeconomic status among children aged 3-6 years.

## Materials and methods

Study design and population

The study population consisted of 260 children aged 3-6 years, attending the Department of Pedodontics and Preventive Dentistry at Rajasthan University of Health Sciences College of Dental Sciences, Jaipur, India. The inclusion criteria required the children to fall within the specified age range (3-6 years), with or without the presence of carious lesions, and to be accompanied by a parent or legal guardian capable of providing informed consent. The presence or absence of ECC was subsequently determined through clinical examination in accordance with the World Health Organization (WHO) criteria. Children with developmental dental anomalies, with physical or mental disabilities, or whose guardians refused participation were excluded.

A cross-sectional observational study was conducted to assess the relationship between ECC and BMI in preschool-aged children. The research adhered to standardized clinical and anthropometric assessment protocols and was executed within a pediatric dental outpatient department setting.

The sample size was calculated based on the following equation: $\text{Sample size}=\text{z}^{2}\times\left(\text{p}\right)\times\left(1-\text{p}\right)/\text{e}^{2}$. Here, z is the critical value of the normal distribution at the required confidence level, p is the sample proportion, and e is the margin of error (5%). Hence, $\text{n}=1.96^{2}\times0.20\times0.80/0.05^{2}=246$. Thus, the minimum sample size required was calculated to be approximately 246.

To account for a possible 10% non-response or attrition rate, the sample size was inflated: $\text{Adjusted sample size}=246/0.90=273$. However, due to logistical and operational feasibility, the final sample size was set at 260 children aged 3-6 years, which still meets the power requirements for the study.

Based on a confidence interval of 95%, a type I error of 5%, and a prevalence of 20% based on previous studies [11], the sample size was estimated to be 236. Taking a 10% attrition rate, the sample size was increased to 260.

Ethical consideration

The study protocol was reviewed and approved by the Ethics Committee of Rajasthan University of Health Sciences College of Dental Sciences (approval number: EC/PG-07/2021). Informed written consent was obtained from the parents or legal guardians of all participating children, in accordance with the ethical standards of the Declaration of Helsinki.

After ethical clearance, the study proceeded with the enrollment of 260 eligible children, whose demographic distribution is summarized in Table 1.

**Table 1:** Distribution of the study subjects Demographic and clinical characteristics of the study population (N=260): this table presents the distribution of the study subjects by gender, age, and presence of ECC, highlighting the baseline characteristics of the sample population aged 3-6 years. ECC: early childhood caries

Study subjects	N	Percentage
Gender		
Male	152	58.5
Female	108	41.5
Age (in years)		
3	64	24.6
4	59	22.7
5	72	27.7
6	65	25
Caries status		
ECC absent	171	65.8
ECC present	89	34.2

Clinical examination

**Figure 1:**
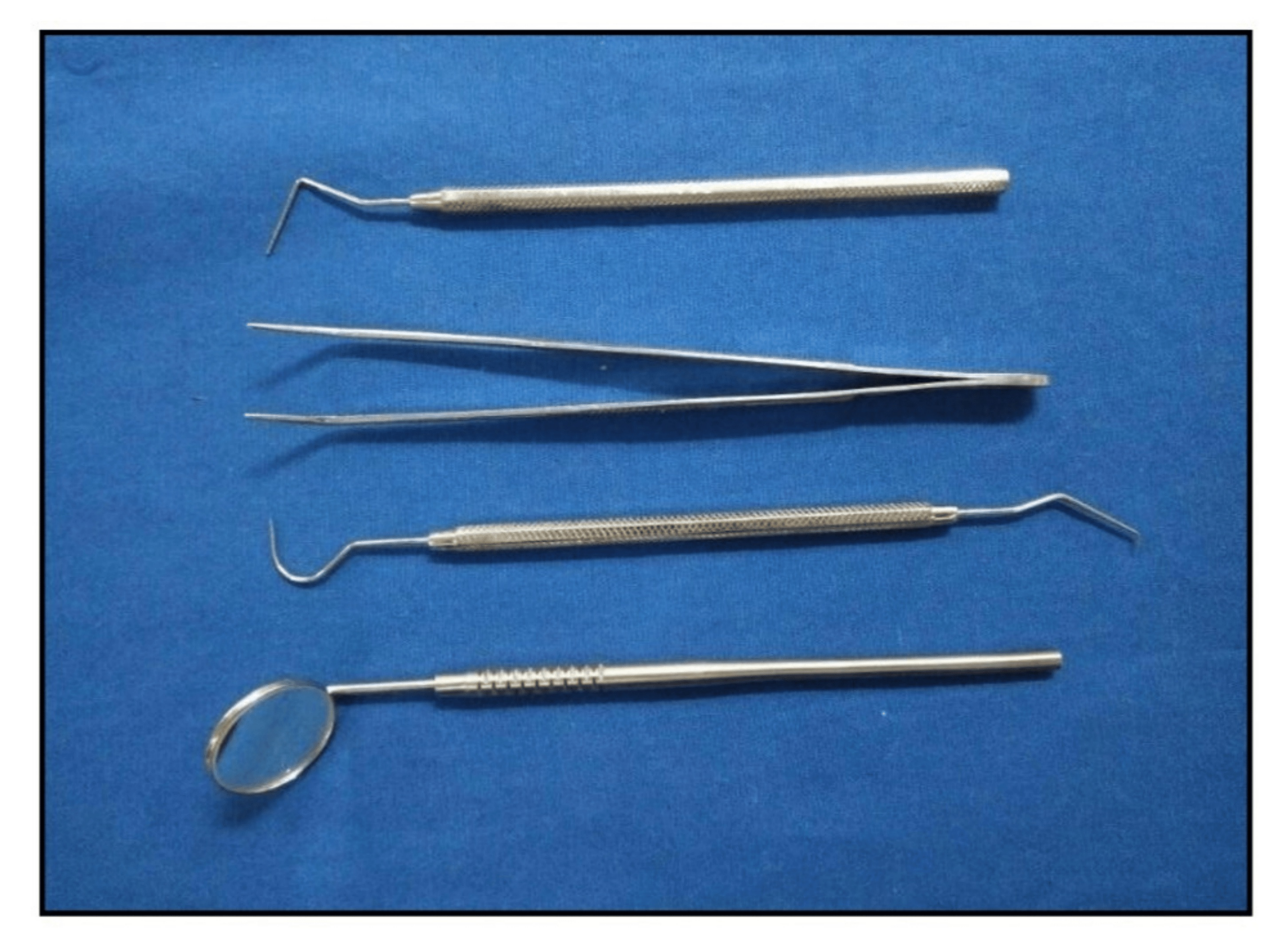
Diagnostic armamentarium

Under the guidance of a senior faculty member, the first author (Surbhi Sharma), a postgraduate trainee in pediatrics, performed the clinical examination. Reliability was confirmed through pre-study inter- and intra-examiner testing using Cohen's kappa, which produced a value of 0.87, indicating excellent agreement. To ensure consistency, all evaluations were conducted by a single calibrated examiner. The decayed, missing, and filled teeth indices (DMFT (Decayed, Missing, Filled permanent Teeth)/deft (decayed, extracted, filled primary (baby) teeth)) were used to measure dental caries in compliance with WHO standards [12]. A weighing machine and stadiometer were used to record weight and height in order to compute BMI, in addition to oral examinations. Throughout the process, standard infection control protocols were closely followed, which included the use of face masks, disposable gloves, and cleaned instruments (Figure 1).

BMI

**Figure 2:**
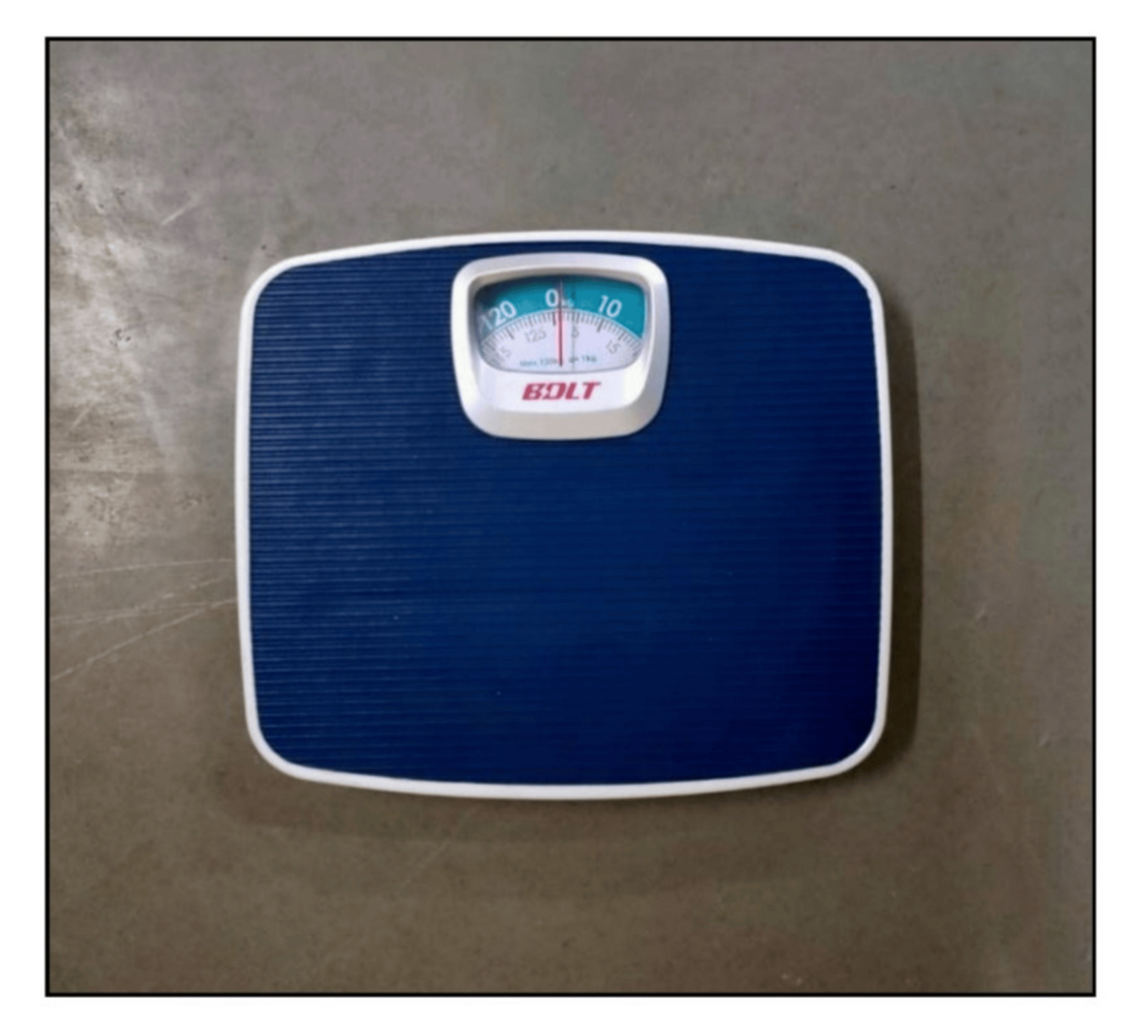
Weighing machine for weight measurement

**Figure 3:**
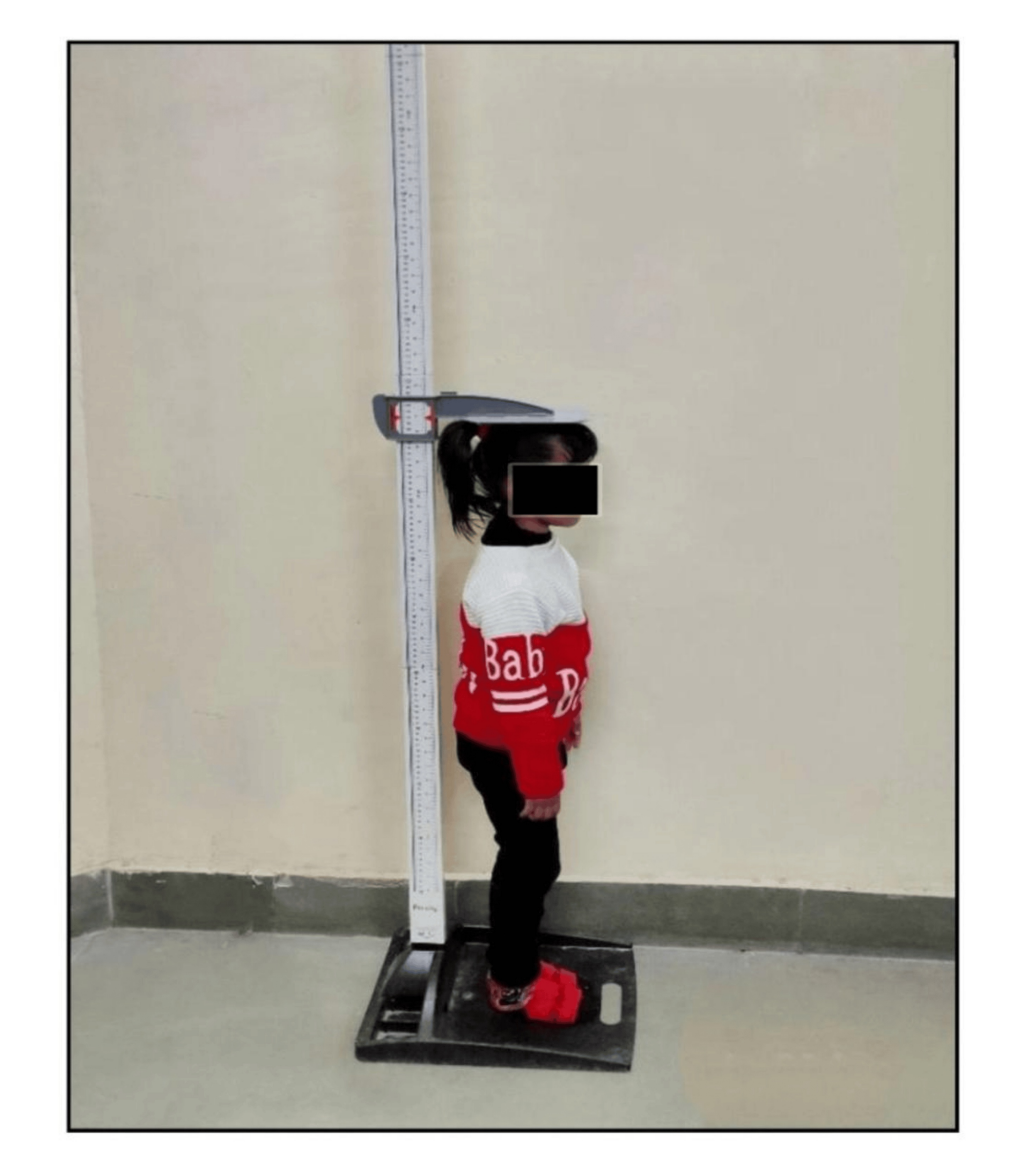
Stadiometer for height measurement

BMI was calculated by entering the recorded weight (Figure 2) and height (Figure 3) of the subjects into the Centers for Disease Control and Prevention (CDC) online Child and Teen BMI Percentile Calculator. BMI-for-age percentiles were used to categorize the children following CDC guidelines: those below the 5th percentile were considered underweight, 5th to less than 85th percentile as normal weight, 85th to less than 95th percentile as overweight, and at or above the 95th percentile as obese [10].

CDC growth charts [10] were used as a standard reference for the BMI of boys and girls. To simplify the comparison of the BMI of the subjects, the following data were extracted from the above CDC growth charts for the BMI of boys and girls between two and 20 years of age and converted into tabular form (Tables 2-3) according to the percentile criteria as given by CDC.

**Table 2:** BMI of a boy child between three and six years of age BMI classification ranges for boys aged 3-6 years based on CDC percentile standards. BMI: body mass index; CDC: Centers for Disease Control and Prevention

S. no.	Age (in years)	Underweight (according to BMI)	Normal (according to BMI)	Overweight (according to BMI)	Obese (according to BMI)
1	3	<14.3	14.3-17.3	17.3-18.25	>18.25
2	4	<14.01	14.01-16.9	16.9-17.83	>17.83
3	5	<13.5	13.5-16.8	16.8-18.22	>18.22
4	6	<13.4	13.4-17.6	17.6-19.64	>19.64

**Table 3:** BMI of a girl child between three and six years of age BMI classification ranges for girls aged 3-6 years based on CDC percentile standards. BMI: body mass index; CDC: Centers for Disease Control and Prevention

S. no.	Age (in years)	Underweight (according to BMI)	Normal (according to BMI)	Overweight (according to BMI)	Obese (according to BMI)
1	3	<14	14-17.2	17.2-18.26	>18.26
2	4	<13.7	13.7-16.8	16.8-18.04	>18.04
3	5	<13.5	13.5-16.8	16.8-18.22	>18.22
4	6	<13.42	13.42-17.06	17.06-18.8	>18.8

Statistical analysis

The data for the present study were entered into Microsoft Excel 2007 (Microsoft Corporation, Redmond, Washington, United States) and analyzed using IBM SPSS Statistics for Windows, Version 23.0 (IBM Corp., Armonk, New York, United States). The descriptive statistics included frequency and percentages. The level of significance for the present study was fixed at 5%. The intergroup comparison for the difference in frequency between independent groups was done using the chi-squared test.

Nature of Data

The data comprised categorical variables, including the presence or absence of ECC, age, gender, and BMI categories (underweight, normal, overweight, and obese).

Descriptive Statistics

Frequencies and percentages were calculated to describe the distribution of study participants by age, gender, caries status, and BMI categories.

Test of Association

The chi-squared test was applied to assess associations between ECC and independent variables (age, gender, and BMI categories). Chi-squared tests were performed using raw frequency counts of ECC presence/absence across categories (age, gender, BMI). Percentages were calculated only for descriptive reporting, not for statistical testing. A p-value of <0.05 was considered statistically significant. The variables in this study, including ECC status, BMI categories, age, and gender, were categorical in nature. Normality testing is applicable only for continuous data; hence, it was not relevant here. Instead, frequencies and percentages were calculated to describe the data. The chi-squared test, a non-parametric method, was appropriately used to assess associations between categorical variables. Therefore, the analysis did not require normality checks.

## Results

In this study, the distribution of ECC was analyzed in relation to age, gender, and BMI. The intergroup comparison for the difference in frequency between independent groups was done using the chi-squared test. Among the 260 children assessed, ECC prevalence varied modestly by age group: 32.8% of three-year-olds, 42.4% of four-year-olds, 29.2% of five-year-olds, and 33.8% of six-year-olds exhibited ECC. However, this variation was not statistically significant (χ²=2.614; p=0.454). Regarding gender, 34.9% of males and 33.3% of females had ECC, a difference also found to be statistically non-significant (χ²=0.065; p=0.874).

Table 4 demonstrates a lack of statistically significant differences in ECC prevalence across age and gender groups, indicating that these variables may not independently influence ECC distribution in this cohort.

**Table 4:** ECC distribution by age and gender Association between ECC and demographic variables (age and gender) using the chi-squared test analysis. ECC: early childhood caries

Description of the study subjects	ECC absent (%)	ECC present (%)	Chi-squared value (χ²)	P-value	Significance
Age (in years)					
3	43 (67.2%)	21 (32.8%)	2.619	0.454	Non-significant
4	34 (57.6%)	25 (42.4%)			
5	51 (70.8%)	21 (29.2%)			
6	43 (66.2%)	22 (33.8%)			
Gender					
Male	99 (65.1%)	53 (34.9%)	0.015	0.901	Non-significant
Female	72 (66.7%)	36 (33.3%)			

In contrast, the association between ECC and BMI categories showed statistically significant differences. Among underweight children (mean BMI 11.58), 49.4% had ECC. This prevalence was notably lower in children with normal weight (mean BMI 15.01), where 27.3% had ECC. The rates dropped further to 23.1% among both overweight (mean BMI 17.28) and obese (mean BMI 18.37) children. This trend indicated a significant association between lower BMI and higher ECC prevalence (χ²=14.136; p=0.001), highlighting a potential link between undernutrition and increased dental caries in early childhood.

Table 5 reveals a statistically significant association between ECC and BMI, particularly underscoring the heightened prevalence of ECC among underweight children compared to their peers who are normal, overweight, or obese, where the rates are considerably lower and similar.

**Table 5:** Cross-tabulation BMI and ECC status Association between BMI categories and ECC status among children aged 3-6 years, indicating a statistically significant correlation. BMI: body mass index; ECC: early childhood caries

BMI categories	Mean BMI score	ECC present (%)	ECC absent (%)	Total	Chi-squared value (χ²)	P-value	Significance
Underweight	11.58	38 (49.4%)	39 (50.6%)	100%	14.136	0.003	Significant
Normal	15.01	39 (27.3%)	104 (72.7%)	100%			
Overweight	17.28	6 (23.1%)	20 (76.9%)	100%			
Obese	18.37	3 (21.4%)	11 (78.6%)	100%			

## Discussion

The present study demonstrates a significant positive association between ECC and underweight status among children, aligning with a growing body of literature suggesting a complex, bidirectional relationship between oral health and nutritional status. Untreated dental caries can have a profound impact on children's overall development, particularly by influencing growth patterns such as BMI, weight, and height.

One mechanism explaining this association is the pain and discomfort caused by ECC, which may limit a child's ability to chew properly and reduce appetite, leading to inadequate nutritional intake [10]. Moreover, chronic dental infections and inflammation can activate systemic immune responses, diverting energy and metabolic resources away from growth [13]. This is especially concerning in populations where nutritional deficiencies already exist, compounding the risk of stunted growth and underweight.

Our findings are consistent with studies conducted in various socioeconomic and geographic contexts. In the Philippines, Benzian et al. [13] found untreated severe dental decay to be a significant, independent predictor of low BMI among 12-year-old children. Similarly, a randomized controlled trial by Monse et al. [14] showed improvements in weight and BMI following dental treatment in underweight Filipino children, reinforcing the link between oral health and growth.

Additionally, Oliveira et al. [15] explored the correlation between caries and nutritional status in Brazilian children, noting that children with ECC were more likely to be undernourished. Their findings, supported by Soares et al. [16], point toward both direct effects, such as difficulty eating, and indirect effects, like social and economic barriers to accessing both oral and general healthcare.

Socioeconomic disparities further mediate this relationship. Studies like those by Elamin et al. [3] and Knoblauch et al. [8] highlighted how lower socioeconomic status contributes to both a higher prevalence of ECC and an increased risk of undernutrition due to limited access to balanced diets and health services.

The role of diet and socioeconomic conditions in moderating the ECC-BMI relationship is further emphasized by Bahuguna et al. [17], who reported that children from lower socioeconomic backgrounds consuming high-sugar diets had both a higher prevalence of dental caries and a tendency toward altered BMI outcomes. Furthermore, Hooley et al. [18], in a systematic review, found conflicting evidence across studies, suggesting that the association between caries and BMI is likely influenced by multiple interacting factors, including nutrition, socioeconomic status, and oral hygiene behaviors.

In light of this evidence, ECC should be viewed not merely as a localized dental issue but as a systemic health concern with the potential to hinder physical development. Preventive strategies must address not only caries management but also broader determinants of health, including nutrition, education, and access to healthcare services.

Limitations

Although the study employed a strong design with standardized methodology and calibrated examination, certain limitations should be acknowledged. First, being a hospital-based cross-sectional study, the findings may not be fully generalizable to the broader community, as children who do not seek dental care were not represented. The relatively modest sample size, restricted to a single geographic location, may further limit external validity. In addition, the cross-sectional nature of the study precludes establishing causal relationships between ECC and BMI. Furthermore, while clinical examinations followed WHO guidelines, the absence of radiographic assessment might have led to the underestimation of caries prevalence. Lastly, potential confounding variables such as dietary patterns, parental education, and socioeconomic status, although considered in the study, may still influence the observed associations.

## Conclusions

The present study highlights a noteworthy association between ECC and underweight status among preschool-aged children. Nearly half of the underweight children examined were affected by ECC, suggesting that poor oral health may coincide with impaired growth outcomes. This finding adds to the growing body of evidence that dental caries should not be viewed in isolation but rather as part of a broader health profile that includes nutrition, growth, and systemic well-being.

However, the cross-sectional design of this study limits the ability to infer causality, and the hospital-based sample may reduce the generalizability of results to the wider community. Therefore, these findings should be interpreted as indicating a potential relationship between ECC and being underweight, rather than as evidence of a direct causal effect. It remains possible that underlying factors, such as dietary habits, socioeconomic conditions, or limited healthcare access, contribute simultaneously to both dental caries and nutritional status.

Despite these limitations, the study carries meaningful implications. Regular dental screening of underweight children could serve as an early warning sign for broader health concerns, while education programs for parents may encourage healthier dietary practices and improved oral hygiene. Integrating oral health promotion with pediatric nutrition initiatives could be particularly valuable, especially in socioeconomically disadvantaged populations where both malnutrition and caries are prevalent.

Looking ahead, further research, particularly well-designed longitudinal and interventional studies, is needed to confirm the direction and strength of the ECC-BMI relationship, identify causal mechanisms, and evaluate whether integrated preventive strategies can lead to measurable improvements in both dental and general health outcomes. Such efforts will help strengthen the evidence base and guide the development of comprehensive, multidisciplinary child health programs.
